# Identification of Novel Loci Involved in Adalimumab Response in Crohn’s Disease Patients Using Integration of Genome Profiling and Isoform-Level Immune-Cell Deconvoluted Transcriptome Profiling of Colon Tissue

**DOI:** 10.3390/pharmaceutics14091893

**Published:** 2022-09-07

**Authors:** Mario Gorenjak, Gregor Jezernik, Martina Krušič, Pavel Skok, Uroš Potočnik

**Affiliations:** 1 Center for Human Molecular Genetics and Pharmacogenomics, Faculty of Medicine, University of Maribor, Taborska ulica 8, 2000 Maribor, Slovenia; 2 Department of Gastroenterology, University Clinical Centre Maribor, 2000 Maribor, Slovenia; 3 Faculty of Chemistry and Chemical Engineering, University of Maribor, Smetanova ulica 17, 2000 Maribor, Slovenia; 4 Department for Science and Research, University Clinical Centre Maribor, Ljubljanska ulica 5, 2000 Maribor, Slovenia

**Keywords:** Crohn’s disease, adalimumab, transcriptome, isoforms, deconvolution

## Abstract

Crohn’s disease is a consequence of dysregulated inflammatory response to the host’s microbiota. Although anti-TNF treatment improves the quality of the patient’s life, a large proportion of patients lose response to the treatment. The past decade of research has led to a continuum of studies showcasing the heterogeneity of anti-TNF response; thus, the aim of the present study was to dissect transcriptome-wide findings to transcript isoform specific levels and combine the analyses with refined information of immune cell landscapes in colon tissue, and subsequently select promising candidates using gene ontology and genomic integration. We enrolled Slovenian Crohn’s disease patients who were naïve with respect to adalimumab treatment. We performed colon tissue RNA sequencing and peripheral blood mononuclear cell DNA genotyping with a subsequent contemporary integrative approach to combine immune cell deconvoluted isoform transcript specific transcriptome analysis, gene ontology layering and genomic data. We identified nine genes (*MACF1*, *CTSE*, *HDLBP*, *HSPA9*, *HLA-DMB*, *TAP2*, *LGMN*, *ANAPC11*, *ACP5*) with 15 transcripts and 16 variants involved in the adalimumab response. Our study identified loci, some of which were previously shown to contribute to inflammatory bowel disease susceptibility, as novel loci involved in adalimumab response in Crohn’s disease patients.

## 1. Introduction

A dysregulated inflammatory response to the host’s microbiota is a key etiology of Crohn’s disease and ulcerative colitis, and leads to chronic inflammation [1,2,3]. Crohn’s disease incidence in Europe is as high as 12.7 per 100,000 persons per year, or 322 per 100,000 persons, making this disease far from rare [4]. Established explanatory concepts of Crohn’s disease have shown a greater role for genetic predisposition as opposed to environmental factors (cigarette smoking and antibiotic use) in comparison to ulcerative colitis [5,6]. The basis of the chronic inflammatory process is now assumed to be commensal microbiota and dysbiosis [7,8,9]. In addition to mucosal barrier disorder, various defects of bacterial recognition, autophagy, endoplasmatic reticulum stress and immune cells exert an effect on antimicrobial defense and alter the microbiome [9,10]. Subsequently invading bacteria induce the inflammatory response, which is thought to be dysregulated [11]. With tumor necrosis factor alpha (TNF-α) being a key cytokine of inflammation, anti-TNF therapies such as infliximab and adalimumab have started to dominate the treatment of Crohn’s disease [12,13]. Anti-TNF therapy significantly improves the quality of patients’ lives, but a large proportion of patients do not respond to anti-TNF therapy or lose the response after the initial benefit [12,14,15,16]. In recent years, novel biological agents such as vedolizumab, ustekinumab and risankizumab in the treatment of Crohn’s disease have emerged [17,18,19,20]. Even after the advent of novel biological drugs targeting other cytokines, adalimumab and its biosimilars remain widely used biological therapy in inflammatory bowel disease [21]. Recently it was also shown that treatment choices for Crohn’s disease must be individualized for each patient, starting with either infliximab with azathioprine or adalimumab as a first-line therapy for the induction of clinical remission [22].

Almost a decade of research has led to a continuum of studies showcasing the heterogeneity of the anti-TNF response [23,24,25,26,27,28,29,30,31,32]. However, most genomic markers for anti-TNF response in Crohn’s disease do not reach sufficient thresholds and, therefore, no reproducibility between genetic and expression data exists [33]. Recent approaches have identified new anti-TNF candidates using integrative transcriptomic-genomic analyses while accounting for deconvoluted immune cell composition in bulk RNA samples from peripheral blood monocytes [30]. Moreover, it was recently shown that deconvolution of whole-tissue gene expression data yields refined information concerning the immune cell landscape in inflammatory bowel disease [34]. Nevertheless, to date, genetic studies of anti-TNF research in Crohn’s disease focused merely on genes, with disregard to specific isoforms; therefore, the aim of the present study was to:

1. Perform isoform-level transcriptome profiling of colon tissue.
2. Combine transcriptome profiling with refined information concerning immune cell landscapes in colon tissue.
3. Stringently select promising gene candidates based on gene ontology analysis.
4. Integrate and functionally annotate the findings from genome profiling data.

## 2. Materials and Methods

### 2.1. Enrolled Subjects

We enrolled 84 Slovenian patients of Caucasian Central European ethnicity with Crohn’s disease (55 responders and 29 non-responders) who were naïve in respect to treatment with adalimumab and fulfilled the criteria for adalimumab (Humira, Abbott Laboratories, Chicago, IL, USA) therapy initiation. Crohn’s disease diagnosis was made based on a combination of endoscopic, histological, clinical, biochemical, stool and imaging investigations based on ECCO-ESGAR guidelines [35]. Baseline demographics of the enrolled patients is available in previous study [30]. Inclusion criteria consisted of adverse effects to corticosteroids, refractoriness to corticosteroids and previous loss of response to infliximab, with a mandatory minimum eight-week wash-out period [24]. Exclusion criteria were defined as the presence of stenosis, abscesses, total colectomy, history of murine proteins allergy, active tuberculosis or a serious infection in the previous three months, pregnancy, lactation or malignancy, history of *Listeria* infection, HIV, demyelinating disease, chronic viral hepatitis, uncontrolled diabetes mellitus, unstable ischemic heart disease or congestive heart failure, cerebrovascular accidents, history of drug or alcohol abuse, previous treatment with natalizumab or any other conditions which would put the patient at risk based on clinician’s opinion [36]. Initiation of adalimumab treatment consisted of a loading dose of 160 mg followed by 80 mg after two weeks and then a maintenance dose of 40 mg every other week. If the dosage of adalimumab was stable in the last three months, concomitant treatment with azathioprine, 5-amisalycylates, corticosteroids or antibiotics was allowed. Response to the treatment was measured using an IBD questionnaire (IBDQ) after 12 weeks of initiation of the therapy, and response was defined as an increase of the IBDQ score of >22 points after baseline score or a total score of >170 points [37,38]. Healthy (N = 11) and inflamed (N = 11) colon tissue biopsies were also collected for subsequent genetic analyses from 22 enrolled individuals who were followed up 12, 20 and 30 weeks after initiation of the adalimumab therapy and were consistent responders or non-responders.

### 2.2. Extraction of Nucleic Acids

DNA was extracted from peripheral blood mononuclear cells using TRI-reagent (Merck, Darmstadt, Germany) according to manufacturer’s instructions. Before RNA was extracted from colon tissue biopsies, the tissue was first homogenized using a Bullet Blender homogenizer (Next Advance Inc., Troy, NY, USA) followed by immediate extraction of RNA using TRI-reagent (Merck) according to manufacturer’s instructions. Concentration, purity and integrity of nucleic acids were estimated using a Synergy 2 spectrophotometer (Biotek, Winooski, VT, USA) and a 2100 Bioanalyzer Instrument (Agilent, Santa Clara, CA, USA) with an RNA 6000 Nanochip.

### 2.3. Transcript Specific Tissue RNA-Seq Analysis

RNA sequencing was performed on 22 samples with estimated an RIN number >9.0 and a 28S/18S ratio between 1.5 and 2.0. Paired-end RNA sequencing was performed at BGI China (BGI, Shenzhen, China) using a DNBSEQ-G400 apparatus and 2 × 100 bp MGIEasy rRNA depletion kit (MGITech, Shenzen, China) and MGIEasy RNA library prep set (MGITech).

Raw fastq files were first assessed for quality using FastQC software (version 0.11.9, Babraham Bioinformatics, Cambridge, UK) with subsequent trimming of technical sequences using the Trimmomatic tool (version 0.39, USADEL LAB, Aachen, Germany) [39,40]. Transcript specific RNA-seq analysis was performed using a Kallisto pseudoalignment program (Pachter Lab, Berkeley, CA, USA) with GRCh37 reference genome [41]. Estimated counts and tpms were further processed using the R 4.2.1 environment (R Core Team 2020, Vienna, Austria) and using a pipeline described elsewhere [30]. Additional conventional alignment of raw reads to the GRCh37 reference genome was performed to obtain estimated meta-feature raw counts and tpms for deconvolution of immune cells in the bulk RNA from tissue biopsies, as described previously [30,42]. CIBERSORTx (Alizadeh & Newman Lab, Stanford, CA, USA) and an LM22 signature matrix were used to correct for immune cell infiltration in the colon tissue [43].

Transcript specific RNA-seq data analysis was independently performed on healthy tissue samples (four non-responders relative to seven responders) and inflamed tissue samples (seven non-responders relative to four responders). A linear regression model and empirical bayes were fitted and covariate-corrected using four different approaches: (I) corrected for sex, age at diagnosis, corticosteroid use, azathioprine use and aminosalicylates use; (II) corrected for sex, age at diagnosis, corticosteroid use, azathioprine use and deconvolution abundance fraction of T cells; (III) corrected for sex, age at diagnosis, corticosteroid use, azathioprine use and deconvolution abundance fraction of monocytes/macrophages; (IV) corrected for sex, age at diagnosis, corticosteroid use, azathioprine use and deconvolution abundance fraction of dendritic cells.

Results from separate healthy and inflamed tissue analyses were subsequently combined using the MetaVolcanoR R package (version 1.2.0, Cesar Prada, São Paulo, Brasil) to obtain meta-statistically significant differentially expressed transcripts showing the same direction of expression and consistence of perturbation [44]. Additionally, statistically significant differentially expressed transcripts showing opposite direction of expression in healthy and inflamed were also retained for further analyses. Statistically significant differential expression was considered for transcripts with *p* value < 0.05 and Log_2_FC > 2 or < −2.

### 2.4. Gene Ontology Analysis

Gene ontology (GO) analysis was carried out using the clusterProfiler R package (version 4.4, Guangchuang Yu, Guangzhou, China) [45]. Enrichment of terms was performed for biological processes, cellular components and molecular functions. Thresholds were set as *p* value < 0.01 and q value < 0.05.

### 2.5. Genome-Wide Association Analysis

DNA obtained from 84 enrolled patients was genotyped using the genotyping microarray Infinium Global Screening Array (GSA_24v1) (Illumina, San Diego, CA, USA). Quality control, genotype imputation, subsequent association analysis of the obtained genotype data and subsequent integration with transcriptomic data were performed as described previously [30]. Binary logistic Wald regression with adalimumab response at week 12 set as the dependent variable was carried out corrected to age at diagnosis, sex, azathioprine use, use of aminosalycylates, use of corticosteroids and the first four principal components. A statistically significant signal was considered for variants with adjusted *p* value < 0.05. Where more than one variant was present at the same locus, variants were pruned using the SNPclip tool from LDlink software (version 5.4, NIH, Bethesda, MD, USA) with R^2^ set to 0.5 r to obtain independent genomic signals [46]. Regional manhattan plots for gene regions were constructed using LocusZoom [47].

### 2.6. Functional Annotation and eQTL Estimation

Functional annotation was carried out using the HaploReg v4.1 database (accessed on 25 July 2022) [48]. Tissue eQTLs were estimated using the GTExPortal and ENSEMBL database (accessed on 25 July 2022) [49,50]. Transcript-wise eQTLs in our data were calculated using the Kruskal-Wallis H test and were calculated independently for all tissues, healthy tissue and inflamed tissue. A *p* value < 0.05 was considered as a statistically significant eQTL observation.

### 2.7. Construction of Gene Interaction Network and Visualization

Interaction grids of genes of interest were generated and illustrated using the software package CytoScape (version 3.8.2, CytoScape Team) with the integrated application ClueGO (version 2.5.8, Laboratory of Integrative Cancer Immunology (Team 15), Paris, France) [51,52]. To obtain curated data of gene interaction, we expanded our list of genes of interest by adding genes interacting with at least two investigated genes (i.e., genes associated with response to adalimumab) which we obtained from the BIOGRID database (accessed on 28 July 2022) using the biogridR R package (github.com/npjc, Vancouver, BC, Canada) [53,54].

### 2.8. Machine Learning Validation

Validation of identified transcripts and variants was further assessed using machine learning random forest algorithm using the randomForest R package (version 4.6–14, Merck Research Laboratories, Kenilworth, NJ, USA) [55]. Machine learning validation was done using all 15 identified transcripts, transcripts of *LGMN* and *ACP5* genes and three transcripts of the *HLA-DMB* gene. Additionally, validation was made for variants where eQTL association was observed. Results are presented as probabilities after receiver operating characteristics (ROC) analysis using pROC R package (version 1.18.0, Swiss Institute of Bioinformatics, Geneva, Switzerland) [56].

## 3. Results

### 3.1. Transcript Specific RNA-Seq Analysis

Transcript specific RNA-seq data analysis was independently performed on healthy tissue and inflamed tissue. A regression model was fitted and corrected using four different approaches as aforementioned. Deconvolution fractions used in the approaches are presented in Figure S1.

**Figure 1:**
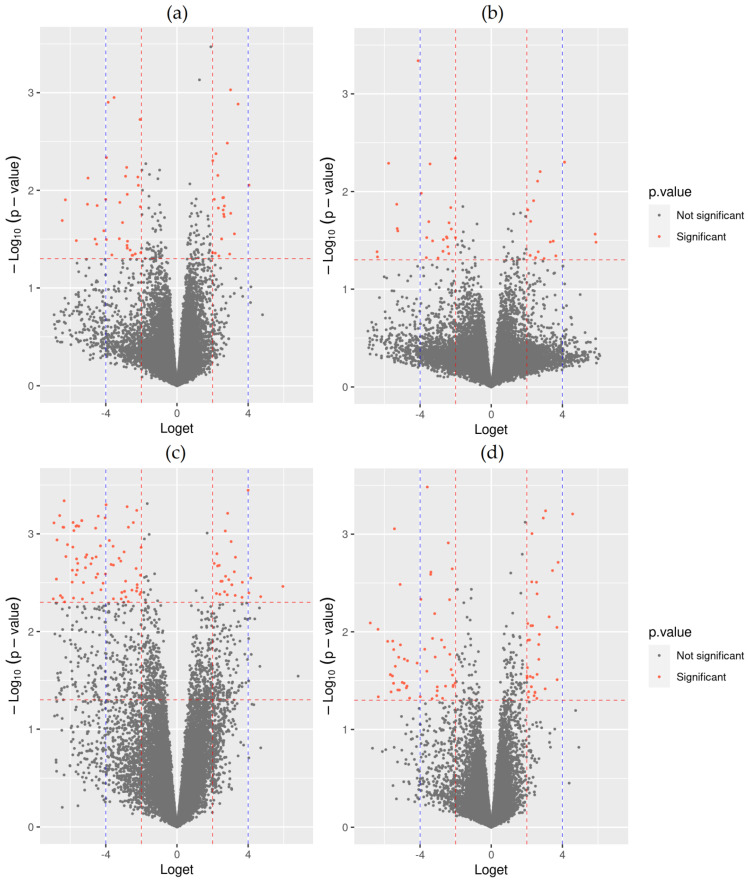
Volcano plots of RNA-seq transcript specific analyses. (**a**) First approach; (**b**) approach corrected for T cells deconvolution fraction; (**c**) approach corrected for monocytes/macrophages deconvolution fraction; (**d**) approach corrected for dendritic cells deconvolution fraction. Red dashed lines indicate borders for statistically significant differentially expressed transcripts.

Using the first approach, we first combined the datasets of the two separate analyses (differentially expressed isoforms (DEIs) between non-responders and responders identified in normal tissue and DEIs between non-responders and responders identified in inflamed tissue) in order to obtain meta-statistically significant DEIs showing the same direction of expression and consistence of perturbation. The analysis identified 62 statistically significant DEIs (Table S1) as evident from Figure 1. Additionally, 26 statistically significant DEIs between non-responders and responders showing the opposite direction in healthy and inflamed tissue were also retained from the two separate analyses (Table S2 and Figure S2).

Using correction for the T cell deconvolution fraction, we observed 52 meta-statistically significant DEIs with conserved perturbation in healthy and inflamed tissues (Table S3) as shown in Figure 1. Thirty-six statistically significant DEIs showing the opposite direction in healthy and inflamed tissue were also observed using the model with correction for T cell fraction (Table S4 and Figure S3).

For determination of meta-statistically DEIs using the regression model with correction for the monocytes/macrophages deconvolution fraction, we used a more stringent threshold (*p* value < 0.005 and Log_2_FC > 2 or < −2), and identified 134 transcripts with a conserved perturbation in healthy and inflamed tissue as shown in Figure 1 (Table S5). Additionally, four transcripts showed statistically significant differential opposite expression in healthy and inflamed tissue (Table S6 and Figure S4).

Using the deconvoluted dendritic cell fraction for correction of the regression model, we observed 98 meta-statistically significant DEIs with conserved perturbation in healthy and inflamed tissue as evident from Figure 1 (Table S7). We additionally observed 157 isoform transcripts with statistically significant differential expression in the opposite directions in healthy and inflamed tissue (Table S8 and Figure S5).

### 3.2. Gene Ontology Enrichment Analysis

Using the results obtained from approaches of isoform specific RNA-seq data analysis, we performed GO analysis for each set of identified isoform transcripts, and their corresponding genes, separately. GO analysis was performed for biological processes, cellular components and molecular function. The most significant enriched terms for each approach are presented in Table 1, and all enriched terms with corresponding gene ratios and gene IDs are presented in Table S9. Enriched terms are mostly correlated to antigen preprocessing and peptide antigen presentation in relation to MHC class I complexes. Furthermore, cytoplasmic translation, lumenal side of endoplasmatic reticulum membrane, ribosome and endocytic vesicle, are also most enriched terms. Altogether, using all statistically significant enriched terms, there are 111 unique gene IDs presented in Table S10.

### 3.3. GWAs Integration, Gene Selection and Functional Annotation

Genomic regions of ± 100 kbp from 111 genes identified in transcriptomics and subsequent GO analyses were further used to integrate transcriptomic and genomic data for stringent gene selection. Integration revealed 54 statistically significant signals in 9 (*MACF1*, *CTSE*, *HDLBP*, *HSPA9*, *HLA-DMB*, *TAP2*, *LGMN*, *ANAPC11*, *ACP5*) out of 111 selected gene regions after application of empirical autocorrelation adjustment (Table S11, Figures S6–S14). All nine genes were represented with altogether 15 isoform transcripts.

Where more variants at the same loci were present, they were additionally pruned by linkage disequilibrium (R^2^ > 0.5) to obtain independent genomic signals presented in Table 2. Independent variants were further functionally annotated to identify possible function on gene expression through epigenetic modifications, chromatin remodeling or protein/motif binding alternations. It was shown that protein binding and motif changes are listed for variants rs10888633, rs35441329, rs28396806, rs28386947, rs10132165 and rs28454947. For variant rs7761882 identified in *HLA-DMB* and *TAP2* gene regions and rs2229531 in the *ACP5* gene region, enhancer histone marks in immune cells from peripheral blood and motif changes are listed. Moreover, variant rs2229531 is a missense variant and flagged as constrained by GERP and SiPhy-omega algorithms. In the variant rs144948822 DNase experiments, motif changes are listed. Variants in the gene region of *LGMN* were the most annotated variants and are listed at promoter and enhancer histone marks in peripheral blood immune cells, intestinal or colon tissue. In DNase experiments, binding of various proteins and motif changes are listed at these variants. Detailed annotation is available in Table S12.

Variants were further analyzed using the eQTL database (accessed on 25 July 2022). Using the genotype-tissue expression GTEx portal database (accessed on 25 July 2022), only rs10137934 (*LGMN*) and rs2229531 (*ACP5*) were listed as significant eQTLs for identified genes in whole blood, small intestines and colon tissue. All other identified variants were listed as significant eQTLs for other neighboring genes in identified gene regions (Table S13). Interestingly, none of these neighboring genes were statistically significant differentially expressed in transcript specific RNA-seq analyses. To assess the eQTLs transcript-wise in our data, we statistically calculated genotype-transcript expression association separately in all tissues, healthy tissue and inflamed tissue (Table 3). Despite the small sample size, we were able to observe statistically significant eQTL for *LGMN* gene (ENST00000555169.1) and rs58531216 in all tissues (*p* = 0.004) and tendency for association in healthy (*p* = 0.053) and inflamed tissue (*p* = 0.052). Tendency for eQTL association was also observed for the *LGMN* gene (ENST000005551669.1) and rs11621843 in inflamed tissue (*p* = 0.052).

### 3.4. Interactions and Machine Learning Validation of Selected Genes

**Figure 2:**
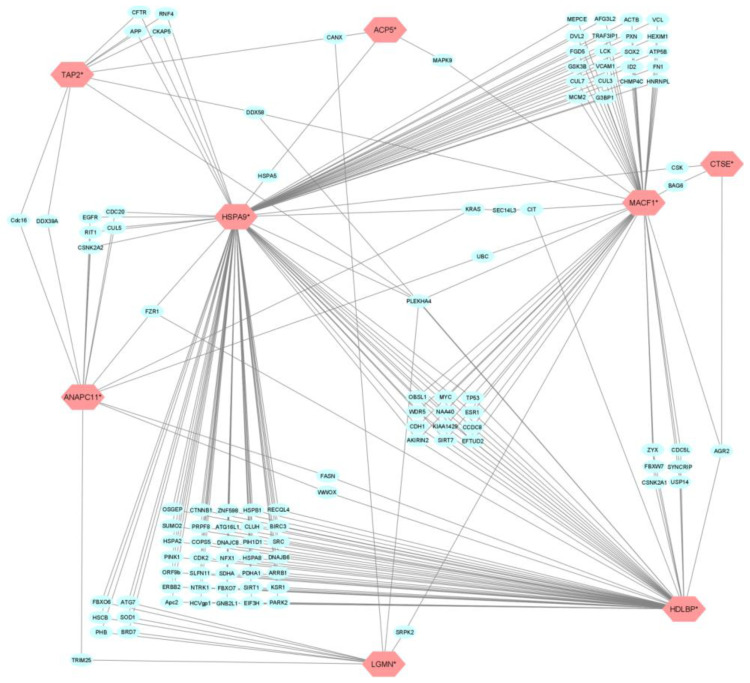
BioGRID network of gene interaction. Red nodes present selected genes. Blue nodes are gene interactors.

The aforementioned nine genes were also assessed for interplay using BioGRID database and visualization of interactions. It was shown that all genes except *HLA-DMB* indirectly interact with each other through a network of auxiliary gene nodes. Interestingly the distance of indirect interactions between target genes is not more than one auxiliary node, as evident from Figure 2.

**Figure 3:**
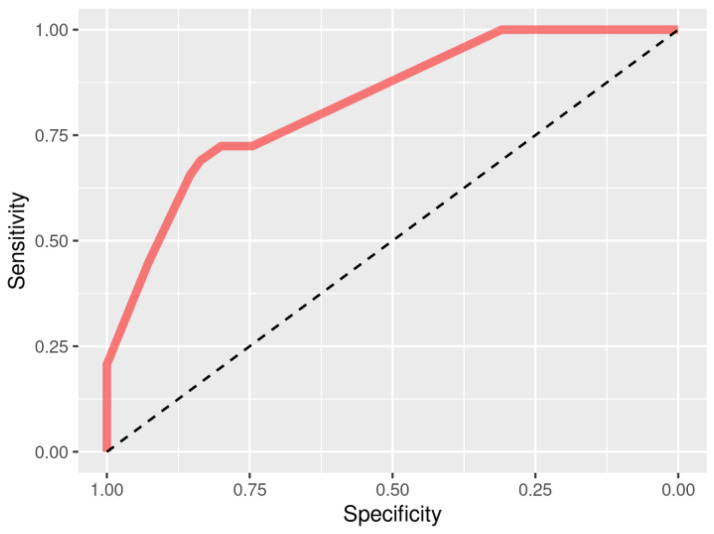
ROC curve analysis for selected variants.

Association of gene expression of the selected transcripts with response to adalimumab was additionally assessed using a Random Forest machine learning algorithm. Assessment was made using the information of tissue state (healthy/inflamed) and expression of: (I) all 15 identified transcripts corresponding to the nine genes; (II) transcripts of *LGMN* and *ACP5* genes with proven eQTLs, and (III) transcripts of *HLA-DMB* gene (ENST00000383231.2, ENST00000428420.2, ENST00000440078.2), which was left out of the BioGRID interactions. All three assessments yielded an AUC of 1 in ROC analyses, thus further confirming the involvement of selected genes in response to adalimumab treatment. Moreover, genotypes from selected variants for which eQTL association was proven (rs58531216, rs10137934, rs2229531) were also assessed for involvement in adalimumab response and yielding AUC of 0.827 (CI95: 0.740–0.913), which further supports the involvement of *LGMN* and *ACP5* genes in the adalimumab response (Figure 3).

## 4. Discussion

The present study investigated colon tissue isoform transcript specific association with response to adalimumab treatment in Slovenian Crohn’s disease patients who were naïve with respect to adalimumab treatment. Using an integrative approach (Figure S15) to combine immune cell deconvoluted isoform transcript specific transcriptome analysis, gene ontology layering and genomic data, we identified nine genes (*MACF1*, *CTSE*, *HDLBP*, *HSPA9*, *HLA-DMB*, *TAP2*, *LGMN*, *ANAPC11*, *ACP5*) with 15 transcripts involved in adalimumab response. Additional genomic integration identified 16 variants residing in aforementioned gene regions associated with adalimumab response. It has been recently shown that deconvolution of whole-tissue gene expression data yields refined information of the immune cell landscape in inflammatory bowel disease [34]. The latter is also supported by previous findings where increased robustness of analyses was shown when deconvolution information was used in regression models [29,42].

Gene *MACF1* was identified as oppositely expressed with one transcript ENST00000372925.2 corresponding to *MACF1* transcript variant 3. The isoform was up-regulated in non-responders relative to responders in healthy tissue, and down-regulated in non-responders relative to responders in inflamed tissue. *MACF1* is also known as *ACF7* and belongs to the spectraplakin family of proteins, which are evolutionarily conserved and are involved in cytoskeletal organization, cell/cell junctions and integrin-mediated epidermal attachments [57]. It was also proven that *MACF1* regulates the molecular mechanism of cytoskeletal coordination and subsequent cell adhesion regulation in intestinal wound repair, and contributes to the development of inflammatory bowel disease [58]. The opposite direction of the expression of *MACF1* transcript between non-responders relative to responders in healthy and inflamed colon tissue additionally suggests an important role of this gene in anti-TNF response in Crohn’s disease.

Two transcripts of the *CTSE* gene corresponding to *CTSE* transcript variant 2 (ENST00000360218.2, ENST00000581049.1-alternate locus) were both up-regulated in non-responders relative to responders. *CTSE* is cathepsin E and belongs to the A1 family of peptidases. *CTSE* was previously identified among the genes, which are uniquely expressed in ulcerative colitis phenotype of the inflammatory bowel disease [59]. Moreover, *CTSE* was also identified in a cluster of the REG4 regulatory network [60]. *CTSE* was also associated with intestinal fibrosis in persistent *Salmonella* infections [61].

Transcript ENST00000310931.4 corresponding to *HDLBP* (high-density lipoprotein binding protein) gene transcript variant 3 was found to be up-regulated in non-responders relative to responders in healthy tissue, and down-regulated in non-responders relative to responders in inflamed tissue. Protein encoded by *HDLBP* binds high-density lipoproteins and regulate excess cholesterol levels in the cells. HDLBP is also known as Vigilin, and has a general function in endoplasmatic reticulum translation and in tumor progression [62]. Furthermore, *HDLBP* was also shown to interact with TCS2 and thus regulates the formation of stress granules [63].

The *HSPA9* gene belongs to the heat shock protein family A. Transcript ENST00000501917.2 belonging to *HSPA9* processed transcript variant 12 was observed to be down-regulated in non-responders relative to responders. It was shown that *HSPA9* plays a role in major up-regulated metabolic pathway during endoplasmatic reticulum stress in colonic goblet cells in a Winnie murine model [64]. Interestingly, *HSPA9* was found to be differentially expressed between steroid responders and non-responders in ulcerative colitis, but did not reach sufficient predictive power to be highlighted [65].

For the *HLA-DMB* gene, three MHC haplotype-specific transcripts corresponding to transcript variant 1 (ENST00000383231.2 MHC haplotype QBL, ENST00000428420.2 MHC haplotype MANN, and ENST00000440078.2 MHC haplotype DBB) were observed to be up-regulated in non-responders relative to responders. *HLA-DMB* is major histocompatibility complex class II beta chain paralogue. This gene is known to be involved in pathways for presentation of viral and self-antigens to T cells and these pathways were found to be unique to the Crohn’s disease phenotype when compared to ulcerative colitis by colon transcriptome [66]. It is also one of the known inflammatory bowel disease genes found to be associated with autoimmune diseases [59]. A previous study also observed that variations within HLA genes had a predominant effect in disease susceptibility as opposed to other genes in MHC gene region [67].

The *TAP2* gene, the neighbor of HLA-DMB, was present with four MHC haplotype specific transcripts corresponding to transcript variant 1. Transcript ENST00000443713.2 MCH haplotype COX was found to be up-regulated in non-responders relative to responders, and other transcripts (ENST00000457634.2 MHC haplotype SSTO, ENST00000452371.2 MHC haplotype DBB and ENST00000455842.2 MHC haplotype APD) were found to be down-regulated in non-responders relative to responders. The discrepancy between the up-regulated and down-regulated state was observed between analyses with correction to T cells and dendritic cell deconvolution fractions, respectively. Interestingly, the up-regulated transcript ENST00000443713.2 MHC haplotype COX was observed only in T cell fraction corrected analysis, and the other three transcripts were down-regulated only in the dendritic cell fraction corrected analysis. The later further proves the complexity of anti-TNF response heterogeneity. *TAP2* is a transporter 2, ATP binding cassette subfamily B member and is a mediator of viral and auto-antigen immunity by pumping cytosolic peptides into the endoplasmatic reticulum for MHC-I-mediated antigen presentation [66]. It was also noted that the expression of *TAP2* was elevated in inflammatory bowel disease in comparison with the controls [66]. It was also shown that the *TAP2* locus may play an important role in Crohn’s disease heterogeneity and steroid responsiveness [68]. Additionally, the *TAP2* gene was identified as a member of whole blood gene panel to distinguish Crohn’s disease and ulcerative colitis [69]. On the other hand, *TAP2* was also identified as a tissue-related common gene for ulcerative colitis in an integrative study of transcriptome-wide association analysis and mRNA expression profiles [70]. Moreover, *TAP2* was one of the identified differentially expressed genes between biopsies from ulcerative colitis patients who were naïve with respect to the treatment and healthy biopsy samples [71].

Legumain (*LGMN*) gene transcript variant 20 ENST00000555169.1 was found to be down-regulated in non-responders relative to responders. *LGMN* encodes a cysteine protease with strict specificity for hydrolysis of asparaginyl bonds, and is involved in processing of the bacterial peptides and endogenous proteins for MHC class II presentation in the lysosome and endosome. Expression of *LGMN* occurs after monocytes differentiate into dendritic cells. In relation to Crohn’s disease, the *LGMN* gene was previously mentioned in a group of highly variable genes between resident and inflammatory macrophage clusters in a study in which the authors identified a cellular module named GIMATS, which consisted of activated dendritic cells, inflammatory macrophages, activated T cells, IgG plasma cells, activated fibroblasts and endothelial cells [32]. GIMATS module was associated with failure to achieve durable remission without corticosteroid use upon initiation of any anti-TNF therapy [32]. To further strengthen the role of macrophages in the response to anti-TNF therapy, it was shown that CD14+ macrophages play a significant role in anti-TNF refractory patients through heightened IL-23 production, which, in turn, leads to binding to the IL23R bearing on TNFR2+ gut CD4+ T cells and results in subsequent STAT3 activation induction leading to expansion of apoptosis resistant intestinal T cells [31]. However, in contrary to our findings, in the present study we did not observe any statistically significant differentially expression of *TNFR2* or *IL23R* genes. Furthermore, the monocyte to macrophage differentiation-associated gene was previously identified as a key player in the adalimumab response [30]. These findings further support the *LGMN* gene as an important gene in the anti-TNF response, as it plays a role in a cell subtype associated with the anti-TNF response. Additionally, concerning the findings that both MHC classes I and II contribute to the Crohn’s disease risk, the present study identified both antigen presentation mechanisms to be important in anti-TNF response [67].

Transcript ENST00000583839.1 corresponding to *ANAPC11* gene transcript variant 19 was identified as down-regulated in non-responders relative to responders. *ANAPC11* is anaphase promoting complex subunit 11 and, to the best of our knowledge, was never before mentioned in relation to inflammatory bowel disease or anti-TNF response. ANAPC11 is a ubiquitination process gene and is associated with dysregulation in innate immunity in malaria [72]. Moreover, *ANAPC11* has been found to be a new independent predictive biomarker for colorectal cancer [73].

Transcript ENST00000218758.5 of *ACP5* gene transcript variant 2 was also down-regulated in non-responders relative to responders. *ACP5* is acid phosphatase 5, tartrate resistant and encodes an iron-containing glycoprotein that catalyzes orthophosphoric monoester to alcohol and orthophosphate. It was identified in a cluster of inflammatory macrophages as a macrophage marker in patients with the GIMATS module of the anti-TNF non-response [32]. *ACP5* also regulates cell cycle progression through mitosis and G_1_, and global hypoacetylation observed in APC mutant yeasts likely leads to chromatin remodeling that can repress expression of genes involved in genomic stability [74].

Integration with genomic data additionally identified 16 variants, which were further functionally annotated and were shown to have a possible impact on gene expression through epigenetic modifications, chromatin remodeling or protein/motif binding alternations. Nevertheless, only three variants have a confirmed eQTL association with aforementioned genes (*LGMN* and *APC5*), and others have a confirmed eQTL with neighboring genes in identified gene regions. Thus, we hypothesize that the epigenetic impact of variants may be extended to the identified gene loci. Furthermore, rs2229531 is an evolutionary conserved missense variant in *ACP5* gene, which may play a role in chromatin remodeling.

It was also observed that the aforementioned genes, except *HLA-DMB*, indirectly interacted with each other through a network of auxiliary gene nodes, where the distance between target genes was not more than one node. To further validate these findings, we applied machine learning probability modeling to obtained data, which further confirmed the role of the aforementioned transcripts and the three eQTL variants in response to anti-TNF therapy responsiveness.

The main limitation of the present study is that we did not have paired healthy-inflamed colon tissues available for our analyses. On the other hand, we acknowledge the homogenous cohort, which was naïve with respect to the anti-TNF treatment, and the use of the only one anti-TNF agent, as strengths in our study.

## 5. Conclusions

Our study used isoform transcript-specific colon tissue transcriptome-wide analysis with subsequent genomic integration, and identified loci, some of which were previously identified to contribute to inflammatory bowel disease susceptibility, as novel loci involved in the adalimumab response in Crohn’s disease patients. As adalimumab is still widely used as a first-line biological treatment, results obtained in the present study may further extent the knowledge of biomarkers for adalimumab treatment response and thus contribute to individualized treatment plans in the evolving field of personalized medicine. Moreover, identified genes aid in better understanding intersections in common molecular pathways of anti-TNF treatment response, and thus may help to discover novel molecular targets for the treatment of Crohn’s disease patients, particularly those who are non-responsive to anti-TNF treatment.

## Figures and Tables

**Table 1:** Gene ontology most significantly enriched terms.

Approach	GO ID	Description	q Value
**Biological Process**			
I consistent *	GO:0002474	Antigen processing and presentation of peptide antigen via MHC class I	9.27 × 10^−6^
I opposite **	NA	NA	NA
II consistent	GO:0048002	Antigen processing and presentation of peptide antigen	0.00018
II opposite	NA	NA	NA
III consistent	GO:0002181	Cytoplasmic translation	4.16 × 10^−17^
III opposite	GO:0019885	Antigen processing and presentation of endogenous peptide antigen via MHC class I	0.00014
IV consistent	GO:0019885	Antigen processing and presentation of endogenous peptide antigen via MHC class I	1.13 × 10^−05^
IV opposite	GO:0039531	Regulation of viral-induced cytoplasmic pattern recognition receptor signaling pathway	0.0019
**Cellular Component**			
I consistent	GO:0071556	Integral component of lumenal side of endoplasmic reticulum membrane	2.91 × 10^−05^
I opposite	GO:0030139	Endocytic vesicle	0.0057
II consistent	GO:0042611	MHC protein complex	0.0015
II opposite	NA	NA	NA
III consistent	GO:0022626	Cytosolic ribosome	2.15 × 10^−16^
III opposite	GO:0071556	Integral component of lumenal side of endoplasmic reticulum membrane	3.25 × 10^−05^
IV consistent	GO:0071556	Integral component of lumenal side of endoplasmic reticulum membrane	0.00049
IV opposite	NA	NA	NA
**Molecular Function**			
I consistent	GO:0003823	Antigen binding	4.39 × 10^−08^
I opposite	NA	NA	NA
II consistent	NA	NA	NA
II opposite	NA	NA	NA
III consistent	GO:0003735	Structural constituent of ribosome	2.24 × 10^−13^
III opposite	GO:0042605	Peptide antigen binding	9.90 × 10^−05^
IV consistent	GO:0042605	Peptide antigen binding	9.83 × 10^−05^
IV opposite	GO:0045296	Cadherin binding	3.44 × 10^−05^

NA: Enriched terms not applicable; * same direction of isoform gene expression change in non-responders vs. responders in healthy and inflamed tissue; ** opposite direction of isoform gene expression change in non-responders vs. responders in healthy and inflamed tissue.

**Table 2:** Independent statistically significant variants.

LOCUS	dbSNP	CHR	BP	*p* Value	q Value	β	SE	AF.RESP	AF.NON
*MACF1*	rs10888633	1	39487164	0.0081	0.0398	1.0167	0.38416	0.44545	0.22414
*MACF1*	rs35441329	1	39537291	0.0030	0.0148	−1.4006	0.47237	0.12727	0.31034
*CTSE*	rs28396806	1	206256869	0.0060	0.0360	−1.3133	0.47774	0.11818	0.31034
*HDLBP*	rs75590598	2	242185654	0.0002	0.0496	−2.3973	0.65155	0.054545	0.24138
*HSPA9*	rs6891007	5	137966643	0.0054	0.0294	−1.0835	0.38979	0.25455	0.46552
*HSPA9*	rs28386947	5	137978163	0.0021	0.0115	−1.4205	0.46251	0.18182	0.41379
*HSPA9*	rs58914268	5	137987814	0.0057	0.0306	−1.0578	0.38237	0.19091	0.39655
*HLA-DMB*	rs7761882	6	32859137	0.0019	0.0443	−1.7556	0.56605	0.1	0.2931
*TAP2* *	rs7761882	6	32859137	0.0019	0.0297	−1.7556	0.56605	0.1	0.2931
*LGMN*	rs58531216	14	93071325	0.0017	0.0158	−1.6627	0.52838	0.072727	0.25862
*LGMN*	rs10132165	14	93091829	0.0036	0.0347	1.4152	0.48635	0.88182	0.7069
*LGMN*	rs11621843	14	93116124	0.0007	0.0071	−1.9507	0.57841	0.054545	0.25862
*LGMN*	rs3814830	14	93118198	0.0042	0.0399	−1.3242	0.46212	0.1	0.2931
*LGMN*	rs10137934	14	93193912	0.0035	0.0339	2.3774	0.81496	0.22727	0.034483
*ANAPC11*	rs144948822	17	79766520	0.0068	0.0136	−3.2032	1.1837	0.0090909	0.12069
*ANAPC11*	rs28454947	17	79769466	0.0214	0.0428	−1.1223	0.48779	0.10909	0.24138
*ACP5*	rs2229531	19	11687195	0.0011	0.0392	−1.96	0.60067	0.081818	0.24138

BP: Base pair location on DNA; β: Beta was calculated for minor allele and response; SE: Standard error; AF.RESP: Minor allele frequency responders; AF.NON: Minor allele frequency non-responders; * Same variant present as above.

**Table 3:** Estimated and calculated transcript eQTLs.

GENE	TRANSCRIPT	dbSNP	eQTL *p* Value ALL	eQTL *p* Value H	eQTL *p* Value D
*MACF1*	ENST00000372925.2	rs10888633	0.084	0.485	0.099
*MACF1*	ENST00000372925.2	rs35441329	1	0.134	0.119
*CTSE*	ENST00000360218.2	rs28396806	0.315	0.439	0.142
*CTSE*	ENST00000581049.1	rs28396806	0.315	0.439	0.142
*HDLBP*	ENST00000310931.4	rs75590598	0.115	1	0.197
*HSPA9*	ENST00000501917.2	rs6891007	0.805	0.319	0.887
*HSPA9*	ENST00000501917.2	rs28386947	0.065	0.102	0.331
*HSPA9*	ENST00000501917.2	rs58914268	0.422	0.077	0.187
*HLA-DMB*	ENST00000383231.2	rs7761882	0.462	0.299	0.339
*HLA-DMB*	ENST00000428420.2	rs7761882	0.462	0.299	0.339
*HLA-DMB*	ENST00000440078.2	rs7761882	0.462	0.299	0.339
*TAP2*	ENST00000443713.2	rs7761882	0.068	0.235	0.218
*TAP2*	ENST00000452371.2	rs7761882	0.181	0.325	0.441
*TAP2*	ENST00000455842.2	rs7761882	0.181	0.325	0.441
*TAP2*	ENST00000457634.2	rs7761882	0.083	0.325	0.339
*LGMN*	ENST00000555169.1	rs58531216	0.004	0.053	0.052
*LGMN*	ENST00000555169.1	rs10132165	0.345	0.134	0.769
*LGMN*	ENST00000555169.1	rs11621843	0.211	0.699	0.052
*LGMN*	ENST00000555169.1	rs3814830	0.355	0.699	0.065
*LGMN*	ENST00000555169.1	rs10137934	0.637	0.617	0.462
*ANAPC11*	ENST00000583839.1	rs144948822	0.501	0.134	0.883
*ANAPC11*	ENST00000583839.1	rs28454947	0.395	1	0.378
*ACP5*	ENST00000218758.5	rs2229531	0.515	0.121	0.624

H: healthy tissue; D: inflamed tissue.

## Data Availability

Data are available on request due to privacy restrictions. The data presented in this study are available on request from the corresponding author.

## Supplementary Materials

The following supporting information can be downloaded at: https://www.mdpi.com/article/10.3390/pharmaceutics14091893/s1, Tables S1–S8. Transcript specific RNA-seq analysis; Tables S9–S13. Gene ontology enrichment analysis; Figure S1. Deconvolution fractions of tissue samples. H: healthy tissue; D: Inflamed tissue; Figure S2. Statistically significant differentially expressed transcripts showing opposite direction in healthy and inflamed tissue in first approach; Figure S3. Statistically significant differentially expressed transcripts showing opposite direction in healthy and inflamed tissue in second approach; Figure S4. Statistically significant differentially expressed transcripts showing opposite direction in healthy and inflamed tissue in third approach; Figure S5. Statistically significant differentially expressed transcripts showing opposite direction in healthy and inflamed tissue in fourth approach; Figures S6–S14. Regional manhattan plot of *ACP5*, *ANAPC11*, *CTSE*, *HDLBP*, *HLA-DMB*, *HSPA9*, *LGMN*, *MACF1*, *TAP2*. Figure S15. Approach and analyses flowchart.
