# Supplementary material for: Identification of Novel Loci Involved in Adalimumab Response in Crohn’s Disease Patients Using Integration of Genome Profiling and Isoform-Level Immune-Cell Deconvoluted Transcriptome Profiling of Colon Tissue
Source: Pharmaceutics. 2022 Sep 7;14(9):1893. doi: 10.3390/pharmaceutics14091893 (PMC9500628; doi:10.3390/pharmaceutics14091893)
Supplement: Supplementary file 1 [file pharmaceutics-14-01893-s001.zip › Supplementary_figures.pdf]

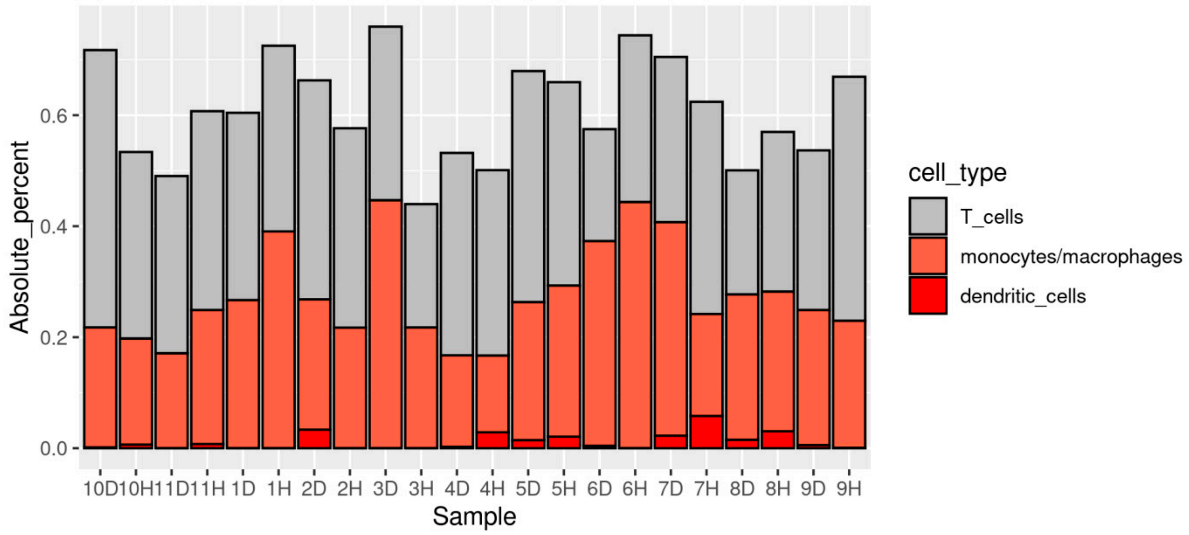

Figure S1. Deconvolution fractions of tissue samples. H: healthy tissue; D: Inflamed tissue.

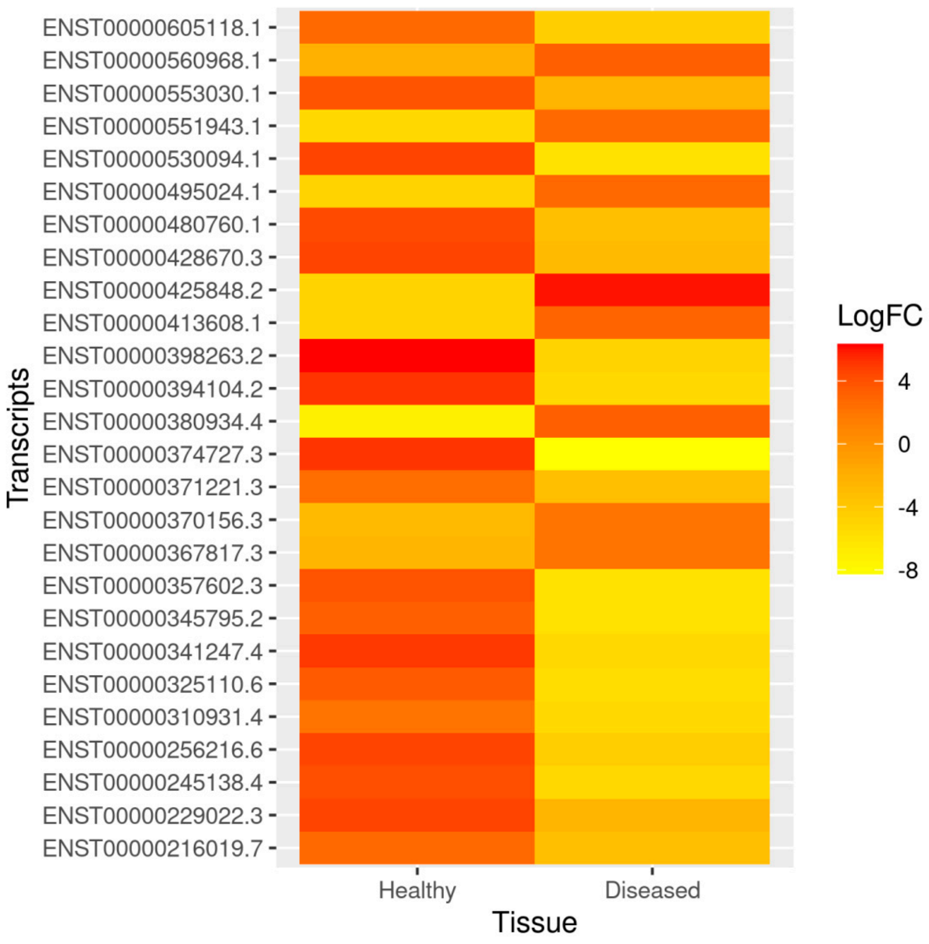

Figure S2. Statistically significant differentially expressed transcripts showing opposite direction in healthy and inflamed tissue in first approach.

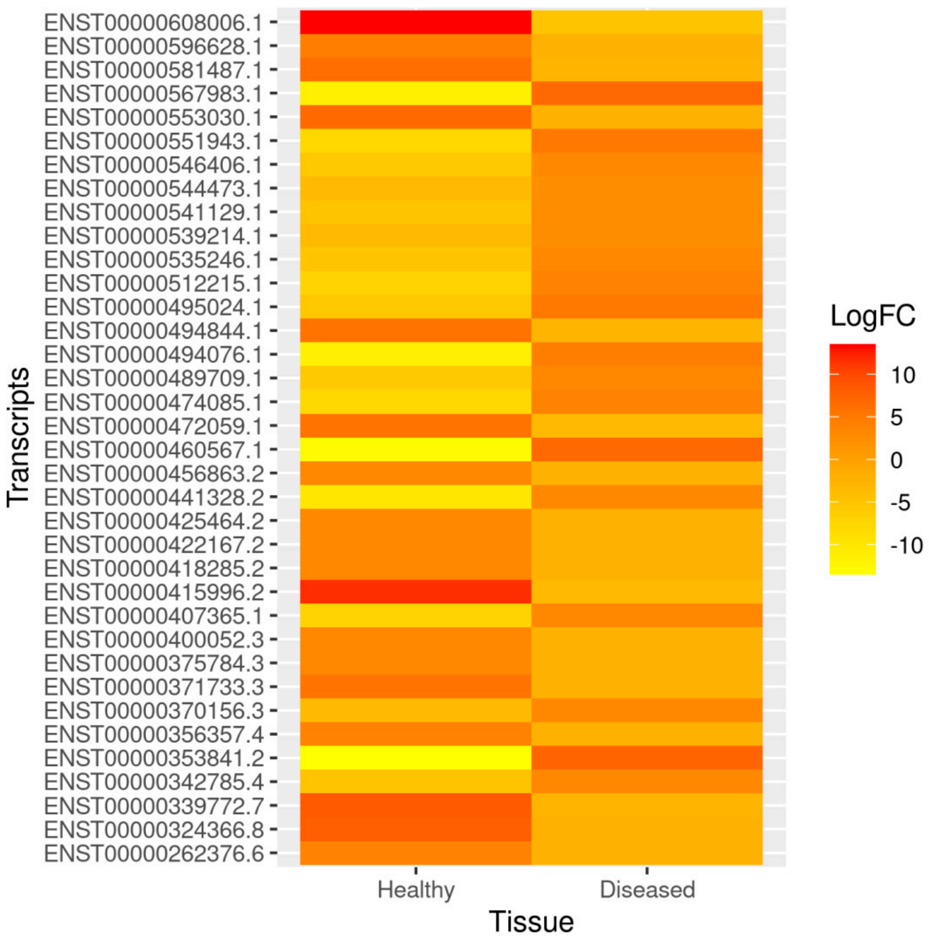

Figure S3. Statistically significant differentially expressed transcripts showing opposite direction in healthy and inflamed tissue in second approach.

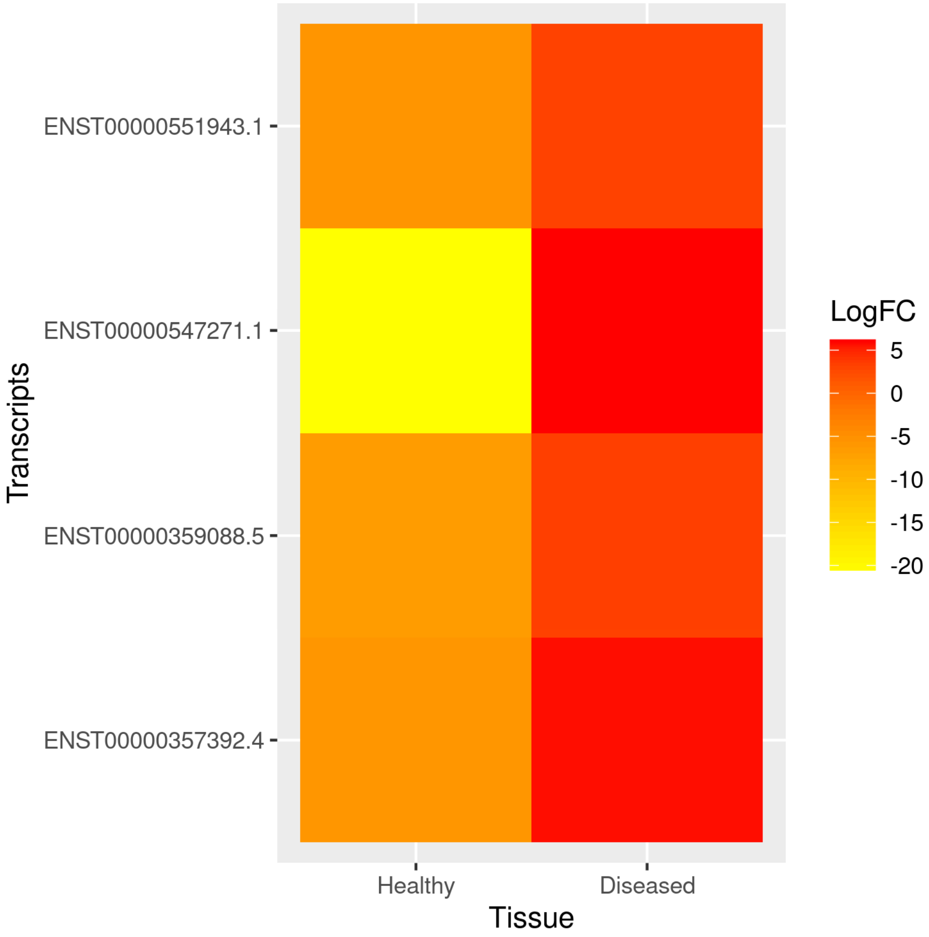

Figure S4. Statistically significant differentially expressed transcripts showing opposite direction in healthy and inflamed tissue in third approach.

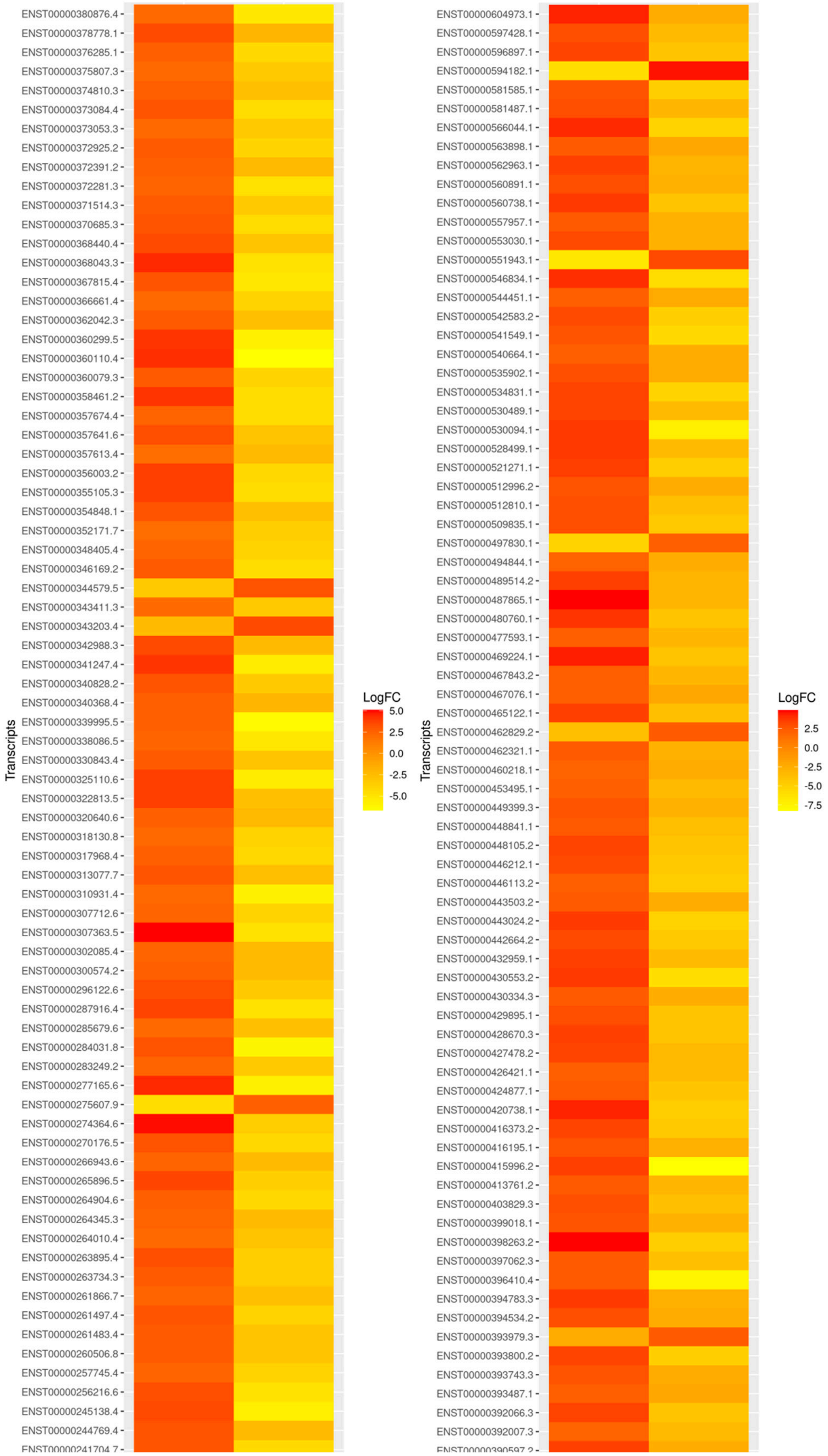

Figure S5. Statistically significant differentially expressed transcripts showing opposite direction in healthy and inflamed tissue in fourth approach.

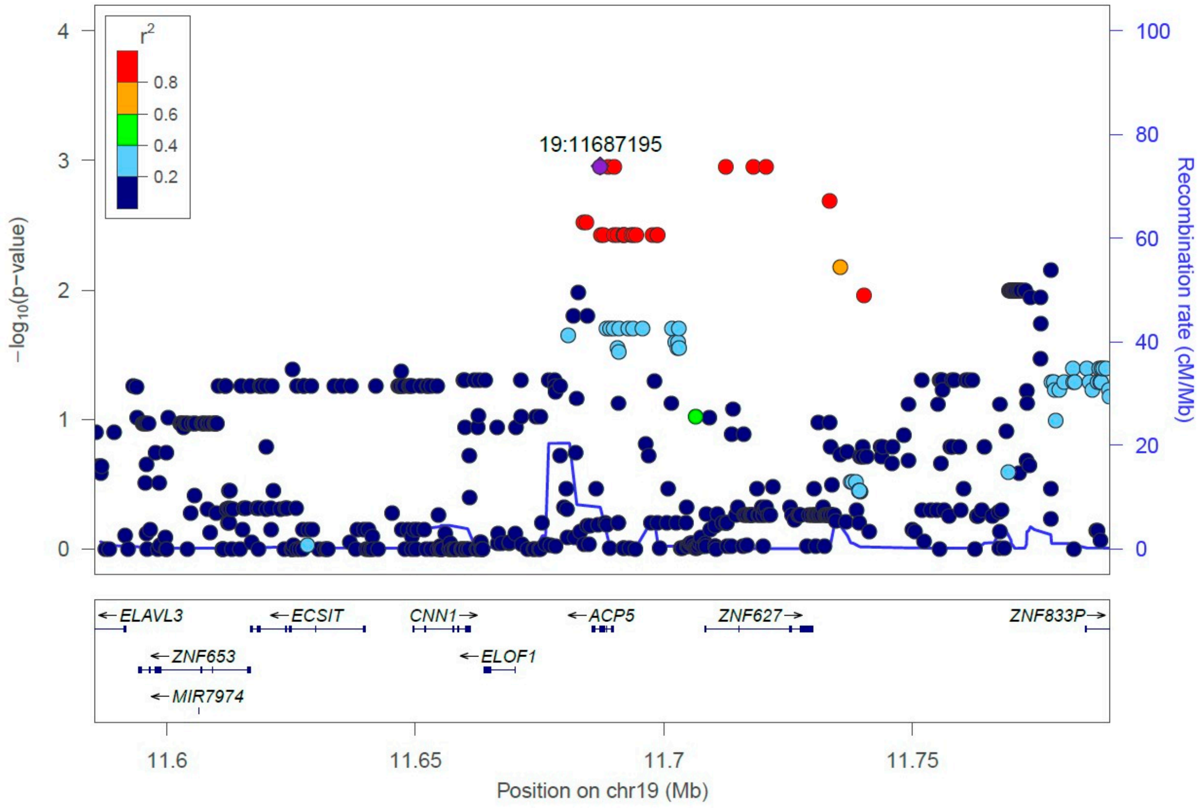

Figure S6. Regional manhattan plot of *ACP5* gene/locus.

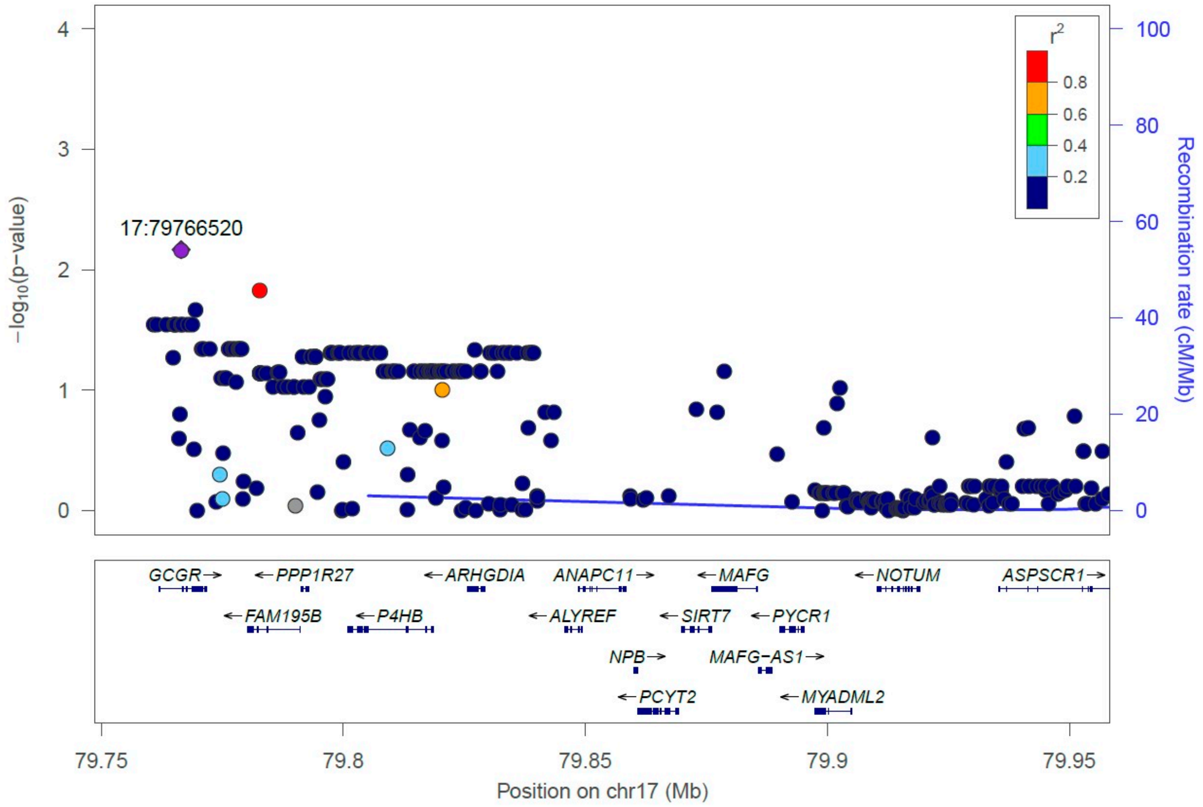

Figure S7. Regional manhattan plot of *ANAPC11* gene/locus.

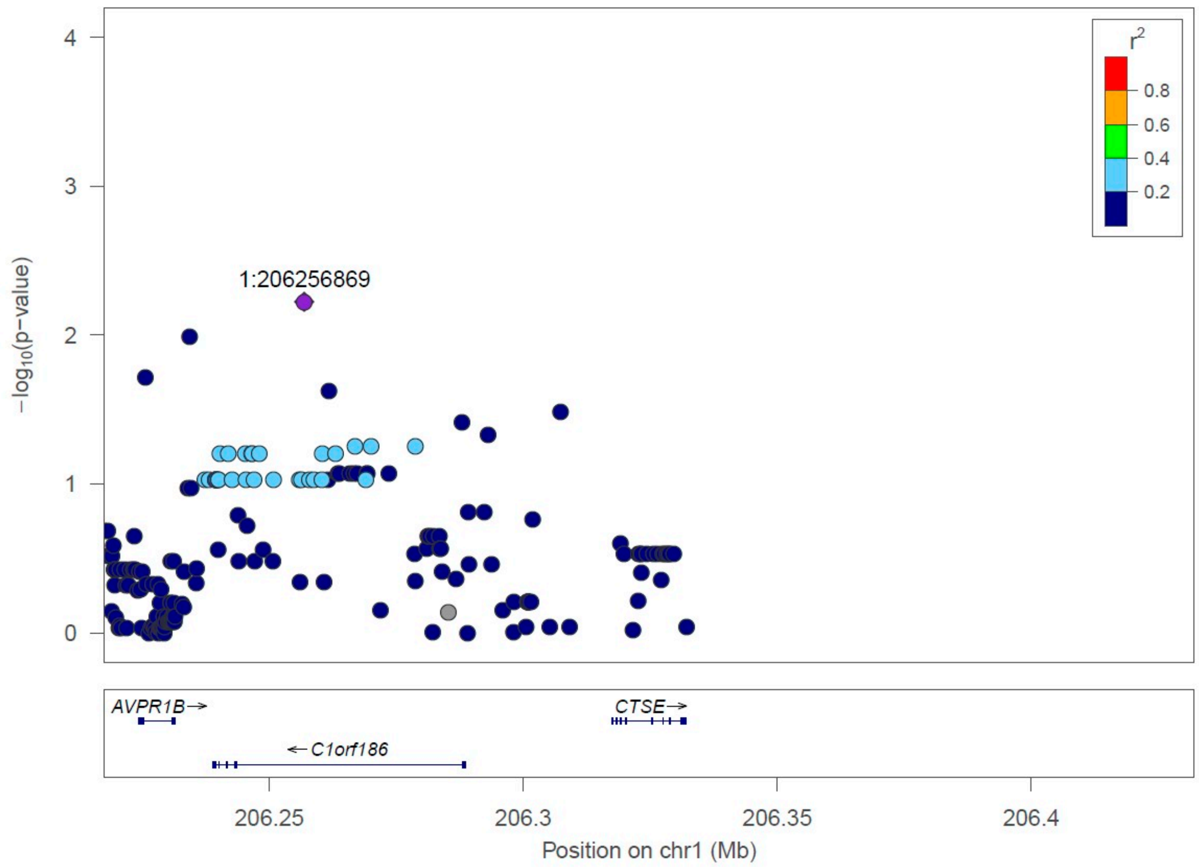

Figure S8. Regional manhattan plot of *CTSE* gene/locus.

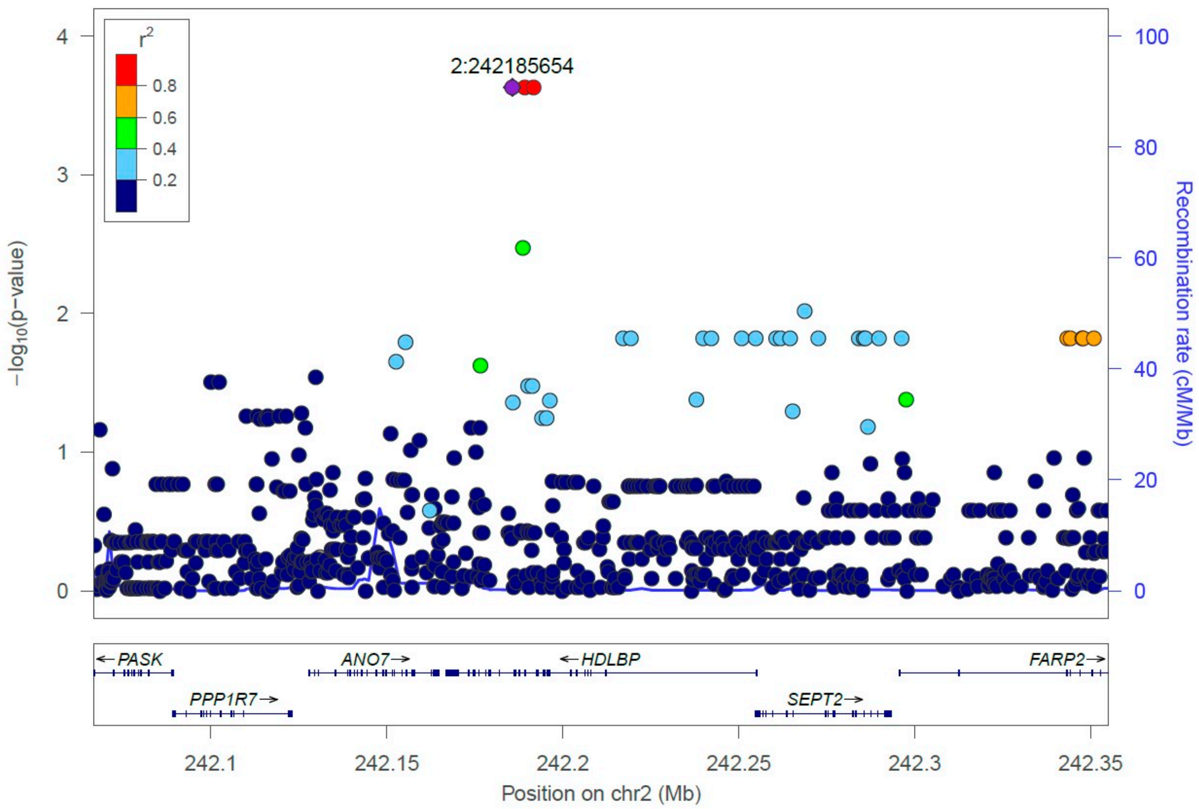

Figure S9. Regional manhattan plot of *HDLBP* gene/locus.

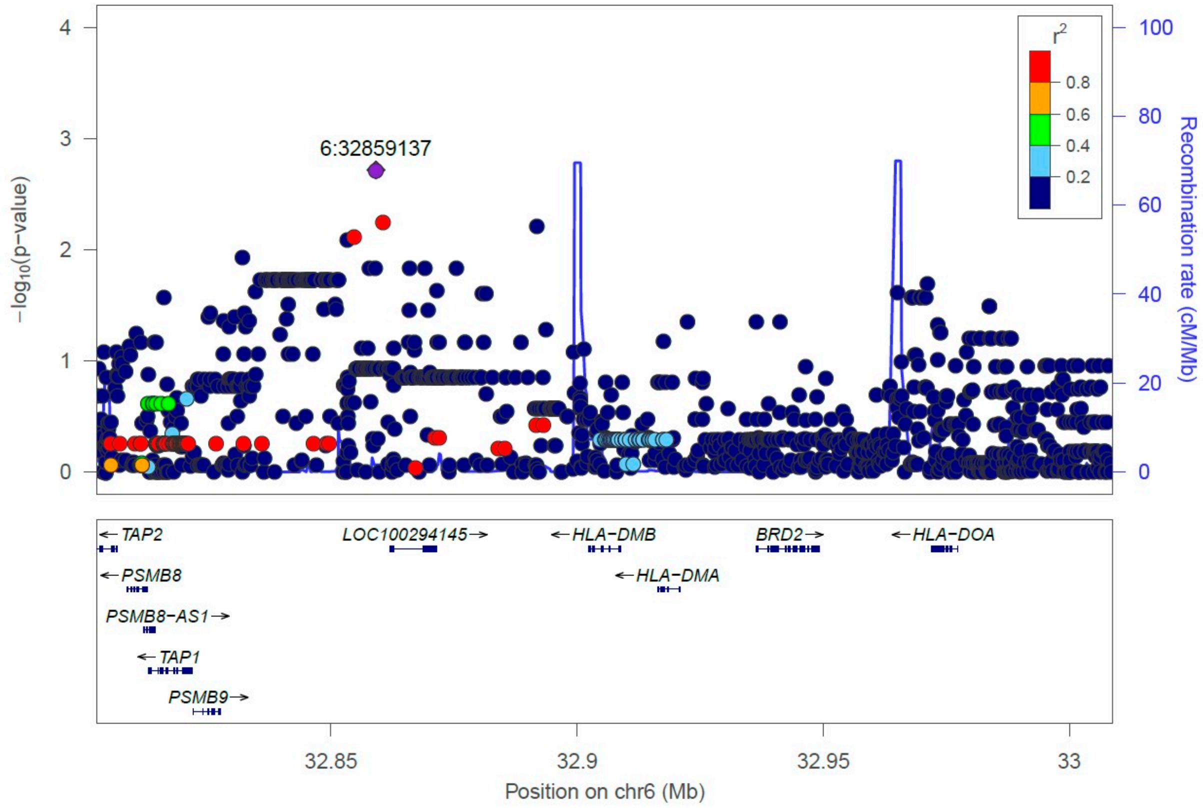

Figure S10. Regional manhattan plot of *HLA-DMB* gene/locus.

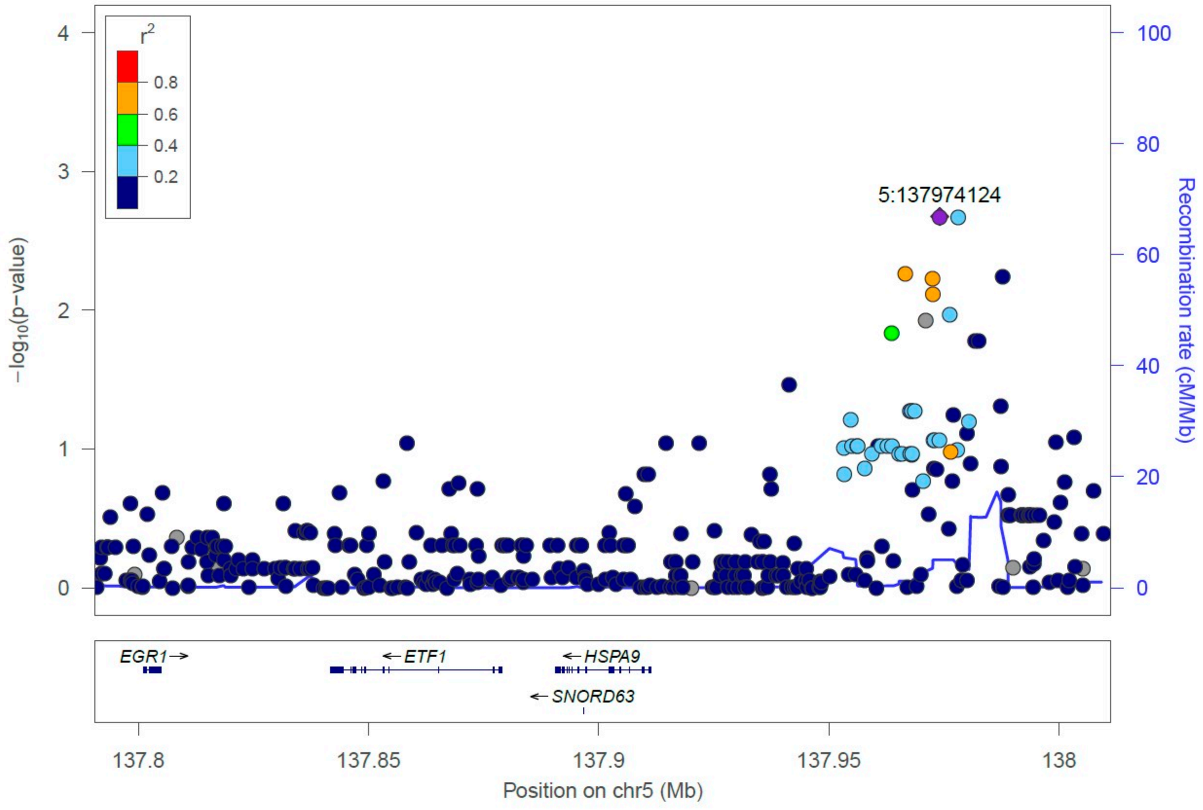

Figure S11. Regional manhattan plot of *HSPA9* gene/locus.

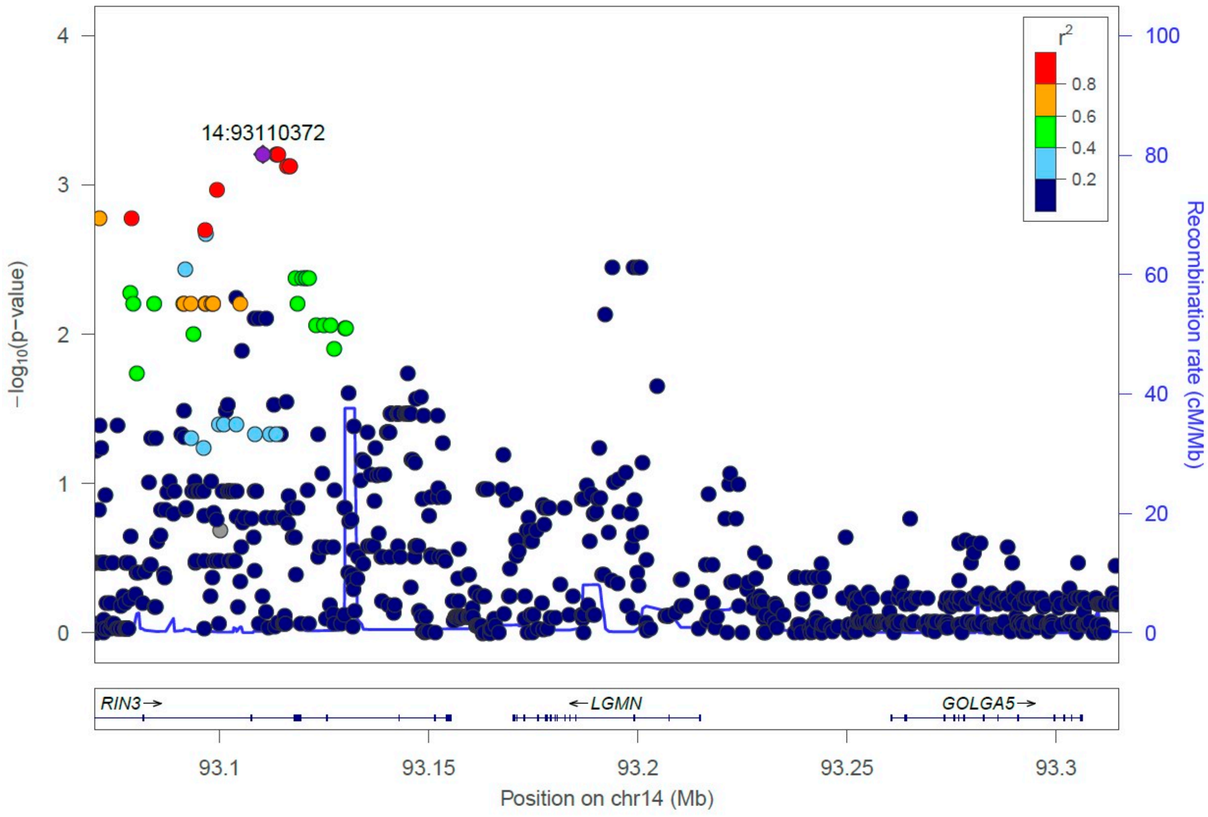

Figure S12. Regional manhattan plot of *LGMN* gene/locus.

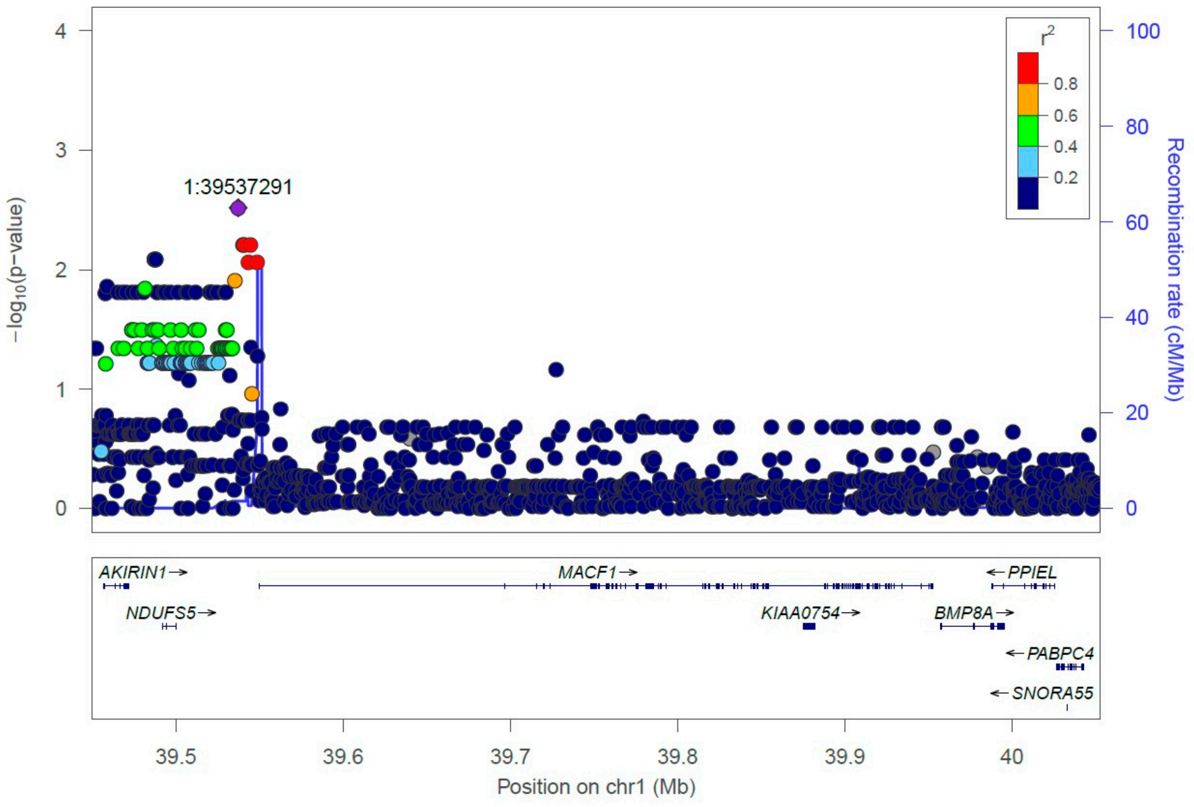

Figure S13. Regional manhattan plot of *MACF1* gene/locus.

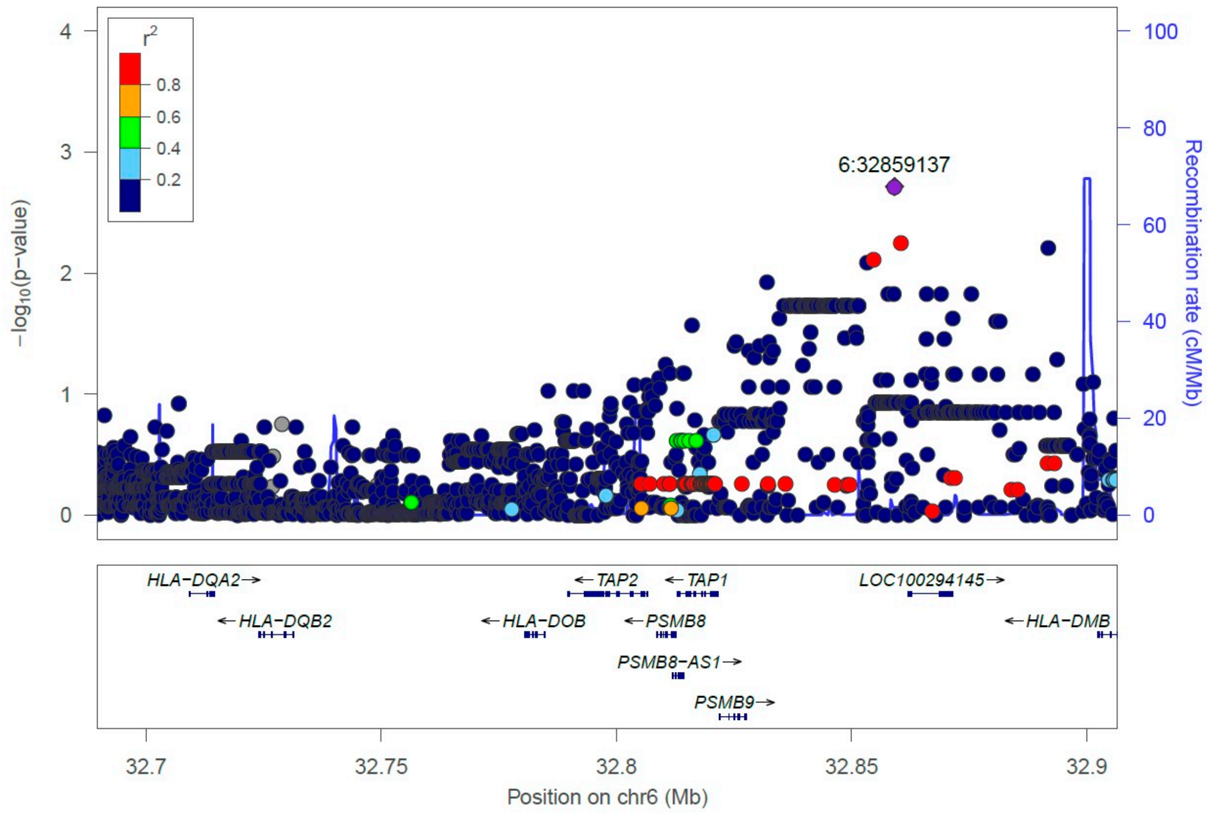

Figure S14. Regional manhattan plot of *TAP2* gene/locus.

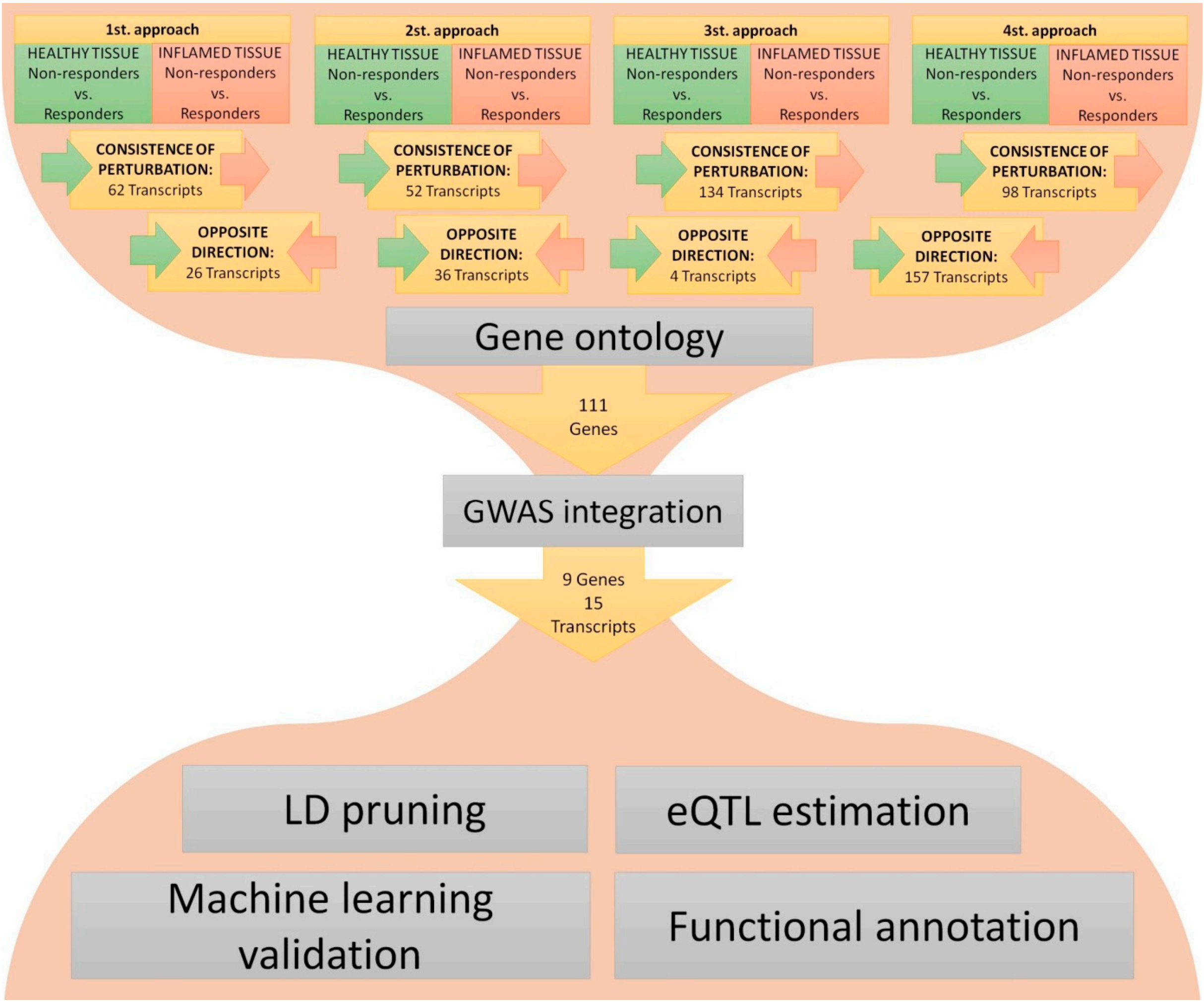

Figure S15. Approach and analyses flowchart.
